# Fetal Alcohol Programming of Subsequent Alcohol Affinity: A Review Based on Preclinical, Clinical and Epidemiological Studies

**DOI:** 10.3389/fnbeh.2020.00033

**Published:** 2020-03-10

**Authors:** Roberto Sebastián Miranda-Morales, Genesis D’Aloisio, Florencia Anunziata, Paula Abate, Juan Carlos Molina

**Affiliations:** ^1^Instituto de Investigación Médica Mercedes y Martín Ferreyra, INIMEC-CONICET- Universidad Nacional de Córdoba, Córdoba, Argentina; ^2^Facultad de Psicología, Universidad Nacional de Córdoba, Córdoba, Argentina; ^3^Instituto de Investigaciones Psicológicas, Facultad de Psicología, Universidad Nacional de Córdoba, Córdoba, Argentina

**Keywords:** fetal alcohol programming, odor and taste, reinforcement, anxiety, associative learning, acetaldehyde, opioid system

## Abstract

The anatomo-physiological disruptions inherent to different categories of the Fetal Alcohol Spectrum Disorder do not encompass all the negative consequences derived from intrauterine ethanol (EtOH) exposure. Preclinical, clinical and epidemiological studies show that prenatal EtOH exposure also results in early programming of alcohol affinity. This affinity has been addressed through the examination of how EtOH prenatally exposed organisms recognize and prefer the drug’s chemosensory cues and their predisposition to exhibit heightened voluntary EtOH intake during infancy and adolescence. In altricial species these processes are determined by the interaction of at least three factors during stages equivalent to the 2nd and 3rd human gestational trimester: (i) fetal processing of the drug’s olfactory and gustatory attributes present in the prenatal milieu; (ii) EtOH’s recruitment of central reinforcing effects that also imply progressive sensitization to the drug’s motivational properties; and (iii) an associative learning process involving the prior two factors. This Pavlovian learning phenomenon is dependent upon the recruitment of the opioid system and studies also indicate a significant role of EtOH’s principal metabolite (acetaldehyde, ACD) which is rapidly generated in the brain *via* the catalase system. The central and rapid accumulation of this metabolite represents a major factor involved in the process of fetal alcohol programming. According to recent investigations, it appears that ACD exerts early positive reinforcing consequences and antianxiety effects (negative reinforcement). Finally, this review also acknowledges human clinical and epidemiological studies indicating that moderate and binge-like drinking episodes during gestation result in neonatal recognition of EtOH’s chemosensory properties coupled with a preference towards these cues. As a whole, the studies under discussion emphasize the notion that even subteratogenic EtOH exposure during fetal life seizes early functional sensory and learning capabilities that pathologically shape subsequent physiological and behavioral reactivity towards the drug.

## Introduction

Two drug-related phenomena act as the foundation for the present review: (i) among other drugs of abuse, ethanol (EtOH) recruits non-associative and associative learning capabilities of the organism; and (ii) age of onset of EtOH-related experiences represents a critical factor determining later use and abuse of this psychotropic agent. Both issues are intrinsically related to the concept of early-life adaptations to environmental disturbances that increase susceptibility to diseases in later life (Barker et al., 2009; Hay, 2009; Calkins and Devaskar, 2011). Under the framework of this vision, we will primarily examine fetal alcohol programming processes, which according to preclinical, clinical and epidemiological studies, represent a critical factor in the structure of subsequent affinity for the drug and the future well-being of the organism. To accrue this goal the present review requires the analyses of the recruitment of early sensory capabilities when the drug invades the prenatal milieu, early EtOH’s motivational properties and the likelihood of learning processes combining both factors. Other critical elements that are needed to understand short- and long-lasting consequences of fetal alcohol programming are related with the critical participation of EtOH’s principal metabolite (acetaldehyde, ACD), how the endogenous system modulates the reinforcing effects of the drug and the effects of the interaction between prenatal and postnatal experiences that shape alcohol recognition, discrimination and preference patterns.

From a general perspective, there is no doubt that EtOH’s teratogenic potential represents the main issue linking embryonic and fetal life with maternal consumption of the drug. The identification of a syndrome (Fetal Alcohol Syndrome, FAS) representing one of the major congenital causes of mental disabilities (Lemoine et al., 1968; Jones et al., 1973) as well as the subsequent recognition and definition of Fetal Alcohol Spectrum Disorders (Riley and McGee, 2005; Riley et al., 2011; Mattson et al., 2019) have guided most of the literature concerning early development and EtOH intoxication. According to the US National Institutes of Health (ncbi.nlm.nih.gov; October 2019), approximately 17,000 articles arise when linking the terms “fetal” and “alcohol.” Within this considerable number of peer-reviewed studies, it is possible to detect an early publication that explicitly examined the consequences of fetal chronic exposure to EtOH upon subsequent intake patterns of the drug (Bond and Di Giusto, 1976). According to these authors, and based on an animal model frequently employed to assess EtOH’s teratogenic properties, voluntary intake of the drug in an altricial species such as the rat, increases as a function of prenatal exposure to this psychotropic agent. There were methodological problems in this study since experimental pregnant rats consumed a liquid diet containing Sustagen (a milk-based supplement) and ethanol while control mothers were fed on lab chow. This obviously implies possible differential nutritional effects that can also lead to ethanol intake changes in the progeny limiting the conclusions of this study. In addition, given the lack of prior information concerning this phenomenon, it was certainly difficult to determine is such an effect was modulated by factors such as alterations in neurotransmitter systems that sensitize the organism to EtOH’s motivational properties (e.g., positive reinforcing effects and/or negative reinforcing effects primarily related with the drug’s antianxiety consequences), comorbidity of neurobehavioral disorders (hyperactivity, attentional deficits, depression, impulsivity), disruptions in basic sensory capabilities that impede or alter olfactory and gustatory discrimination processes, etc. Probably none of these modulators can now be dismissed. It is our expectation that the present review will provide at least partially, notions related with the fact that early experiences with the drug recruit functional capabilities of the organism that result in subsequent alcohol affinity. As will be analyzed this recruitment can be established when employing even subteratogenic levels of EtOH exposure. Through the examination of this phenomenon we hope to provide an expansion of the pre-existing vision of neurobehavioral disorders that need to be prevented, diagnosed or treated in children and adolescents with an early positive EtOH history.

The present review will be organized as follows: (i) a first section, primarily centered on preclinical studies, analyses different experimental strategies alluding to fetal capabilities of altricial mammals related to the detection and discrimination of EtOH’s sensory attributes; (ii) a second item, also based in animal studies, incorporates the notion of EtOH’s reinforcing effects that promote associative learning memories leading to heightened EtOH affinity; (iii) the third section emphasizes the need to consider the drug’s principal metabolite (ACD) as a critical factor modulating EtOH’s reinforcing effects; (iv) the role of the endogenous opiate system modulating prenatal EtOH reinforcement is additionally considered *via* animal behavioral and psychopharmacological studies; and (v) Finally, the last section of the review is devoted to highlighting human clinical and epidemiological studies endorsing the notion that prenatal EtOH experiences have a major impact on the development of subsequent EtOH affinity. As will be observed, this human section has been organized to depict the analogies and homologies existing between animal and human findings.

## Do Fetuses Perceive EtOH’s Chemosensory Properties?

Fetal and neonatal sensory discrimination capabilities have been described in different altricial species (rabbits, sheep, rats) including humans (Lecanuet et al., 1995; Schaal et al., 2002, 2004; Clark-Gambelunghe and Clark, 2015; Fulgione et al., 2017). Neuroethological studies indicate that chemosensory systems rapidly become functional *in the uterus* (Molina et al., 1999, 2007a; Schaal et al., 2004; Bloomfield et al., 2017). This development is required for essential survival purposes related to subsequent maternal attachment processes including the discrimination and recognition of the main nutrients (colostrum and milk) that will be provided both peri- and neonatally (Cernoch and Porter, 1985; Makin and Porter, 1989; Marlier et al., 1998; Miller and Spear, 2009; Díaz-Marte et al., 2010; Corona and Lévy, 2015). In accordance with Nicolaidis (Nicolaïdis, 2008) intrauterine experience with flavors derived from the mother’s diet is swallowed by the fetus generating the activity of a functional olfactogustatory system. First experiences with flavors occur prenatally *via* the accumulation of sensory cues in the amniotic fluid yielding olfactory, gustatory and trigeminal stimulation that is also observed when these cues are present in breastmilk (Mennella and Beauchamp, 1991; Beauchamp and Mennella, 2011; Forestell and Mennella, 2017); phenomena that serve to establish orosensory learning processes that reunite the basic characteristics of a prenatal and/or perinatal imprinting process.

Maternal EtOH consumption also results in the accumulation of the drug in the amniotic fluid as well as in breastmilk (Bachmanov et al., 2003; Molina et al., 2007b). The low molecular weight of this psychotropic agent permits its passage through the placenta and the levels attained in the amniotic fluid and fetal blood are comparable to those existing in maternal plasma (Szeto, 1989; Hayashi et al., 1991; Domínguez et al., 1998). As indicated by Glendinning et al. (2017), fetal perception of the drug’s chemosensory properties in the rat can be established through two non-mutually exclusive pharmacokinetic mechanisms; humoral and intraoral. The presence of the drug in the bloodstream of the immature organism is capable of generating hematogenic stimulation of chemosensory receptors (Molina and Chotro, 1989a,b; Molina et al., 1989) while the presence of EtOH in the amniotic fluid directly stimulates olfactory, gustatory and trigeminal receptors (Glendinning et al., 2017).

At least four reviews have acknowledged the consequences of fetal exposure to the drug’s chemosensory attributes upon later recognition and preference to these cues (Bachmanov et al., 2003; Spear and Molina, 2005; Molina et al., 2007b; Abate et al., 2008). Hence, the present section will only be devoted to summarizing the main findings discussed in such reviews which reinforce the notion that fetal alcohol perception critically intervenes in how the organism later relates to the drug. The following issues will be presented as a function of different experimental strategies analyzing this specific phenomenon.

A first preclinical approach emerges from studies performed in near term rat fetuses (21 gestational days) *via* direct administration of minimal amounts of EtOH in the amniotic fluid (40 μl of a 6% v/v EtOH solution) or a novel scent (lemon). Ten minutes later pups were delivered *via* cesarean surgical procedures (Chotro and Molina, 1990). During the 2nd week of postnatal life, pups were tested in an olfactory preference test as well as in terms of EtOH intake patterns. The sensory cue presented in the amniotic sac was the one preferred in the olfactory evaluation. In addition, pups with a short antenatal EtOH experience were the ones exhibiting heightened levels of EtOH ingestion (Dominguez et al., 1993). Utilizing a similar experimental approach, it was also observed, almost immediately after birth, that pups exposed prenatally to EtOH exhibited clear behavioral and physiological (bradycardia) responses indicative of an orienting response to the smell of the drug (Chotro and Molina, 1992). This antenatal experience, that was not sufficient to generate pharmacologically relevant EtOH levels in the fetus, was also observed to potentiate infantile alcohol-odor conditioned preferences when the odor of the drug was paired with a sweet positive reinforcer (Chotro et al., 1991).

A second experimental approach validating the importance of prenatal exposure to EtOH’s sensory properties has been employed during a fetal stage of brain development in the rat analogous to the 2nd human trimester (Dobbing and Sands, 1979; Tran et al., 2000). During this stage rats and humans exhibit a functional olfactogustatory system (Molina et al., 1999; Bloomfield et al., 2017) as well as significant innervations of target fields of the oral trigeminal system (Mbiene and Mistretta, 1997). The procedure normally involves maternal EtOH intoxication utilizing subteratogenic doses (0.5–2.0 g/kg) that are intragastrically administered during the last 4 days of gestation (gestational days 17–20). This protocol yields comparable EtOH levels in the amniotic fluid, fetal and maternal blood (range according to the doses previously mentioned: 50–160 mg/dl; (Domínguez et al., 1996). The first study utilizing this approach indicated that EtOH was not sufficient to cause teratogenic disruptions related to placenta weights, umbilical cord lengths, offspring’s body weights, weights and/or size of the olfactory bulbs, cerebral hemispheres, and cerebellum. Furthermore, subsequent research indicates that this EtOH exposure procedure does not affect neural migration processes which allow the appropriate synaptic organization of the glomeruli of the principal olfactory bulbs (Pueta et al., 2011). Yet, fetal early experience with EtOH had a dramatic effect upon perinatal responsiveness to the smell of the drug. Pups pretreated with 1.0 or 2.0 g/kg EtOH consistently showed a marked decrement in their motor activity when stimulated with the scent of the drug. This reaction was markedly different from the one caused by a novel citric scent (Domínguez et al., 1996). We later observed that perinatal infusion of EtOH concentrations similar to those encountered in the amniotic fluid also exerted a sedative effect upon a motor activity (Domínguez et al., 1998). This effect was very similar to the one generated by the amniotic fluid in rat pups with no prior history with the drug. The biological fluid had no sedative effects in pups pretreated with EtOH. In other words, fetal alcohol experience determined a similar profile of perinatal responsiveness to EtOH’s sensory cues as the one encountered when control perinates were confronted with a relevant biological sensory cue. Experience with the sensory cues of the amniotic fluid is known to intervene in the acceptance of colostrum and milk (Marlier et al., 1998; Al Aïn et al., 2013; Schaal et al., 2013) or even to act as an appetitive conditioned stimulus (CS; Chotro et al., 2007). These results seem to strengthen the hypothesis of a sensory-imprinting phenomenon generated *via* prenatal EtOH exposure (Nicolaïdis, 2008).

Is this particular prenatal experience sufficient to modify EtOH affinity later in life? The answer is affirmative and once again implies fetal memories relative to EtOH’s chemosensory attributes. During infancy, near term rat fetuses exposed to maternal EtOH intoxication not only ingest higher amounts of EtOH but also show drinking preferences when confronted with the configuration of a sucrose-quinine solution (Domínguez et al., 1998). In genetically heterogeneous rats, this gustatory combination has been demonstrated to act as a psychophysical equivalent relative to the EtOH’s taste (Di Lorenzo et al., 1986; Kiefer, 1995; Bachmanov et al., 2003). It has also been demonstrated that heightened EtOH intake patterns are mainly generated when fetal experience occurs during the last two gestational days (19 and 20). This brief temporal exposure is also capable of increasing EtOH palatability (Díaz-Cenzano and Chotro, 2010).

Rat and human neonates detect even minimal amounts of EtOH in maternal milk [rats: 175 mg/dl following maternal intoxication with 2.5 g/kg EtOH (Pepino et al., 1999); humans: 50 mg/dl following the intake of a single beer (Mennella and Beauchamp, 1991, 1993; Mennella, 1997)]. It has also been demonstrated that in rats, EtOH maternal intoxication (1.0 or 2.0 g/kg) during late pregnancy exacerbates offspring’s consumption of milk contaminated with the above mentioned minimal amounts of the drug without affecting the consumption of the uncontaminated nutrient (Pueta et al., 2008).

The early recognition of EtOH’s sensory attributes as a function of maternal EtOH intoxication during late pregnancy has also been investigated relative to the effects of the drug upon breathing plasticity. Cullere et al. (2015) have observed that brief fetal EtOH experiences in rodents disrupt neonatal breathing patterns when the organism is re-exposed to the toxic effects of the drug. Most importantly, this detrimental effect is exacerbated under the presence of EtOH odor. Similar results have been reported in rats during postnatal days 3–9; a developmental stage that partially overlaps with the 3rd human gestational trimester characterized by a brain growth spurt (Macchione et al., 2016). In subsequent items of this review, these issues will be re-examined as a function of associative learning processes based on EtOH’s sensory cues and the drug’s motivational effects.

The analysis of sensory-related memories emerging from prenatal EtOH experiences has also been addressed through animal models based on the use of moderate to high levels of EtOH throughout most of the gestational period. The most frequent model that has been utilized is known to exert teratogenic effects and implies a progressive increase in the amount of EtOH provided to the mother *via* a liquid diet (Miller, 1992). Based on this animal model, neurophysiological responsiveness to different odorants including EtOH was mapped across the olfactory epithelium. The main findings observed in infants prenatally exposed to the drug indicate a tuned neurophysiological response to EtOH odor despite an altered general response to alternative odorants. This result was also supported by a specific odorant-induced reflexive sniffing response to EtOH odor emerging from a positive prenatal history with the drug (Youngentob et al., 2007; Eade et al., 2010). Chronic prenatal EtOH exposure also resulted in heightened voluntary EtOH intake during early postnatal life and adulthood (Youngentob et al., 2007). When focusing on the gustatory system, fetal EtOH exposure resulted in the taste-mediated acceptability of EtOH and a diminished aversion to the drug’s quinine-like taste component (Youngentob and Glendinning, 2009). In a recent study, the research group of Glendinning et al. (2017) has provided new evidence concerning fetal alcohol exposure reprogramming of the peripheral taste and trigeminal systems. The main findings indicate that prenatal EtOH diminishes aversive flavor attributes of the drug such as its bitter taste and the burning sensation mediated by the trigeminal system.

Sensory persistence of fetal-alcohol related chronic exposure was also assessed during adolescence utilizing an animal social transmission paradigm involving the perception of EtOH odor. The paradigm is based on the interactions between a sober organism (observer) and an intoxicated counterpart (demonstrator) which provides sensory information of the drug *via* direct elimination processes (e.g., alveolar excretion, urination, salivation, et cetera (Fernández-Vidal and Molina, 2004; March et al., 2013d). A positive prenatal alcohol history promoted social interactions with an intoxicated peer and the juvenile re-exposure experience subsequently enhanced EtOH odor response as assessed in a whole-body plethysmograph (Eade and Youngentob, 2009). When configuring the neurophysiological and behavioral studies conducted by Youngentob and Glendinning (2009) the title of one of their articles clearly summarizes the impact of chronic fetal alcohol exposure upon EtOH affinity: “Fetal EtOH exposure increases EtOH intake by making it smell and taste better.”

## A Holistic View of Fetal Alcohol Programming Processes: Is Sensory Familiarization Solely Responsible for Subsequent EtOH Affinity? The Need to Consider Early EtOH’s Motivational Effects

According to the information presented in the preceding item, fetuses detect EtOH’s sensory attributes and memories arising from these early experiences allow latter discrimination of these cues and preference to such stimuli. Is sensory familiarization solely responsible for subsequent EtOH affinity? From a pharmacokinetic perspective, the studies previously reported based on maternal EtOH intoxication imply the juxtaposition of the drug in the amniotic fluid and its presence in fetal blood and brain. Hence, in terms of potential associative learning processes (e.g., pavlovian or classical conditioning), the requirement of temporal contiguity between specific sensory cues and physiological effects of the drug is met.

It can be argued that when animal studies were conducted *via* direct EtOH contamination of the amniotic fluid while avoiding fetal intoxication (Chotro and Molina, 1990, 1992; Chotro et al., 1991) the main phenomenon determining subsequent EtOH recognition and preference obeyed to familiarization with EtOH’s sensory cues. Even under these circumstances, biologically relevant stimuli were found to modulate the memories arising from such experiences. The administration of the drug into the amniotic sac took place only 10 min prior to cesarean delivery. Immediately after delivery, neonatal bodily stimulation was provided to ensure optimal survival rates. This tactile manipulation mimics maternal stimulating effects which, modulated through the endogenous opiate system, acts as an appetitive unconditioned stimulus (US) that is rapidly associated with pre-existing or simultaneously presented olfactory cues (Leon, 1987; Ronca and Alberts, 1994; Roth and Sullivan, 2006; Raineki et al., 2010). Indeed, when considering EtOH administration into the amniotic fluid and its contingency with activating tactile stimulation, it was observed that sensory preferences were highly dependent upon optimal temporal contiguity between these factors (Molina et al., 1999). In other words, the phenomenon under consideration complied with the notion of the acquisition and retention of a conditioned response.

There are numerous examples that endorse the capability of the fetus to learn conditioned responses when pairing a relatively neutral stimulus (e.g., an odorant) with different biologically relevant events. Conditioned taste aversions have been reported in near term rat fetuses when pairing a tastant (apple juice) with the emetic effects of lithium chloride. These aversions are manifested in multiple ways: avoidance of maternal nipples scented with such tastant or spending less time over shavings that contain this gustatory cue (Stickrod et al., 1982) as well as a noticeable delay to traverse a runaway scented with apple to gain access to the mother (Smotherman, 1982). Acute hypoxia induced by clamping the umbilical cord can also lead to odor conditioned aversions (Hepper, 1991) while on the contrary, cessation of hypoxia leads to odor conditioned preferences (Hepper, 1993).

EtOH can be considered as a drug that depending on factors such as dose, age, genetic predisposition, comorbidity with other neurobehavioral disorders, etc. can exert differential triphasic motivational effects. EtOH intoxication promotes positive and antianxiety effects as well as aversive consequences (Pautassi et al., 2009). Positive reinforcing effects play a significant role in the initiation, maintenance of alcohol intake, seeking behavior of the drug as well as in patterns of abuse of this psychotropic agent. These processes can also be modulated by that the drug’s amelioration of aversive states such as anxiety and dysphoria. The progressive maturation of the hepatic system allows the accumulation of peripheral ACD which exerts both gastrointestinal distress and sedative effects known to act as aversive stimuli (Cunningham et al., 2000). Each of these motivational effects can support associative learning given its temporal association with interoceptive or environmental CSs.

When the pregnant female is intoxicated, it is difficult to discern whether fetuses only learn about the sensory components of the drug, its unconditioned effects or the association between these factors. The first animal study that was meant to address these possibilities was originally based on a pharmacokinetic approach involving the distribution of a non-EtOH olfactory cue (cineol, the main component of eucalyptus oil) that easily crosses the placenta and exerts no physiological consequences on the fetus. Cineol can be easily traced in terms of concentration and temporal duration in the amniotic fluid. The strategy was to explicitly associate or not the presence of this CS with the state of fetal EtOH intoxication. These experiences took place during gestational days of 17–20. Three weeks later pups originally exposed to the explicit association between cineol and EtOH intoxication exhibited conditioned orofacial responses when re-exposed to the odorant (Abate et al., 2000). Utilizing a similar strategy, a test was conducted based on the newborn’s first suckling response. The test consisted in the evaluation of neonatal attachment to a surrogate nipple that delivers milk. In pups prenatally exposed to an optimal contingency involving cineol and EtOH intoxication, the presence of the odorant promoted heightened suckling behavior of the surrogate nipple. In addition, this effect was potentiated if neonates were re-exposed to a similar state of intoxication as the one experienced *in utero*; a phenomenon that appears to indicate reactivation of the original associative memory (Abate et al., 2002).

The hypothesis of associative learning mechanisms received additional support through a series of preclinical studies where cesarean delivered pups were tested utilizing two alternative conditioning procedures. The first one utilized the surrogate nipple as a CS paired with an intraoral infusion of EtOH while the second one employed an olfactory CS associated with a similar infusion. Pups exhibited pharmacologically relevant levels of EtOH in blood and in both cases, the presence of the odorant elicited heightened responsiveness to the surrogate nipple (Cheslock et al., 2001). The efficacy of EtOH as a positive reinforcer was also detected when utilizing either intraperitoneal or intracisternal (directly into the cisterna magna) administrations of the drug (Petrov et al., 2003; Nizhnikov et al., 2007).

Taking into account the preceding studies, new experimental approaches were focused on the interaction between prenatal experience with the drug and its subsequent reinforcing effects. Near term rat fetuses were exposed to a moderate EtOH dose and following cesarean delivery they were subjected to paired or unpaired experiences comprising a surrogate nipple and different EtOH doses (0.00, 0.25, 0.50 or 0.75 g/kg) which were intraperitoneally injected. A positive antenatal history with the drug increased the magnitude of appetitive conditioned responses to the nipple mediated by EtOH intoxication and also increased the range of doses yielding a reinforcing effect (Nizhnikov et al., 2006). This phenomenon alludes to a sensitization effect relative to the drug’s motivational properties.

Further evidence indicative of prenatal EtOH sensitization was detected in infants subjected to an operant conditioning procedure where a sweet tastant (sucrose) served as a reinforcer. After operant training, a moderate EtOH dose (0.5 g/kg) was paired with the sweet taste. In pups prenatally exposed to EtOH, this association enhanced sucrose reinforcement and also increased resistance to extinction of the operant response (Culleré et al., 2014). Progressive sensitization to the US facilitates associative learning processes probably by impeding habituation to conditioned signals and strengthening the contingency between these signals and the US (Çevik, 2014). In the case of early EtOH exposure, this entanglement of non-associative and associative learning effects may help to explain the persistence of memories leading to EtOH affinity. Heightened voluntary adolescent EtOH consumption has been detected following brief experiences with the drug during late gestation (Fabio et al., 2015) as well as when drug exposure occurred during most of the gestational period (Eade et al., 2016). In the first case, it was also observed that prenatal EtOH exposure also mitigated the aversive effects of relatively high doses of the drug that are sufficient to generate conditioned taste aversions. Furthermore, late prenatal EtOH exposure has been observed to decrease neural activity within the infralimbic cortex, an area implicated in the extinction of drug-mediated associative memories (Fabio et al., 2013).

Heightened early sensitivity to EtOH’s reinforcing effects has also received support from operant conditioning procedures adapted to the sensory and motor capabilities of the perinate. It was originally demonstrated that perinatal behaviors (nose-poking and front limb movements) deployed to stimulate the mammary gland and to facilitate nipple attachment increase as a function of intraoral delivery of maternal milk (Arias et al., 2007). This technique was adapted to assess the reinforcing value of different EtOH solutions. Operant conditioning mediated by the drug rapidly occurred and perinates exhibited dose-dependent blood EtOH concentrations. At the beginning of an extinction process, pups reinforced with EtOH were also found to exhibit exacerbated seeking behaviors of the drug (Bordner et al., 2008). As assessed through this operant paradigm, late prenatal exposure to EtOH enhances the reinforcing effects of the drug as well as of a taste-related psychophysical equivalent such as the configuration between sucrose and quinine (March et al., 2009). This taste configuration has no reinforcing effects in pups without prior prenatal EtOH exposure. Apparently, the association between EtOH’s sensory attributes and the reinforcing effects of the drug allows the relatively neutral gustatory components later act as second-order reinforcers (Molina et al., 2007a).

Notice that the above-summarized studies seem to indicate that EtOH primarily acts as the appetitive US when employing classical conditioning paradigms or as a positive reinforcer when examining animal operant learning. In some of these studies, EtOH doses yielding relatively high blood and brain levels of the drug were employed. In no case, conditioned taste or odor aversions were detected. These aversions have been observed during infancy, adolescence and adulthood. More specifically, EtOH-mediated conditioned aversions are rapidly established in 10-day-old or older rats (for a detailed ontogenetic revision on this matter see Pautassi et al., 2009). Some studies have utilized high EtOH doses (e.g., 3.0 g/kg) during stages equivalent to the 2nd or 3rd human gestational trimester. In older animals, these doses exert highly aversive effects that seem to provoke emetic-like effects as the ones induced by lithium chloride (Arias et al., 2007; Pautassi et al., 2008). One of these studies was conducted during late prenatal life where pregnant females received binge-like exposures to the drug operationalized through intragastric administration of a 3.0 g/kg dose (peak blood and amniotic fluid levels were approximately equivalent to 200 mg/dl; Chotro et al., 2009). Direct elimination of the drug (e.g., alveolar excretion and salivation) or hematogenic stimulation of sensory receptors promotes associative learning comprising EtOH’s chemosensory cues and the drug’s interoceptive effects (Molina et al., 1989). As described, fetuses exhibit associative learning given the contingency between sensory and toxic effects of EtOH. Dams treated with the above-mentioned dose later exhibited an initial rejection of an EtOH solution in a voluntary intake test suggestive of a conditioned orosensory aversion. On the contrary, their offspring showed alcohol affinity during infancy.

As can be observed, noticeable ontogenetic differences emerge when considering the motivational properties of the drug. Fetuses as well as neonates (7–8 day old rats) treated with a high EtOH dose (3.0 g/kg) later exhibit enhanced drug palatability and heightened predisposition to consume this psychotropic agent. At 10 days of age opposite outcomes (disgust reactions and intake decrements) are detected following toxic experiences generated by a similar dose (Arias and Chotro, 2006). As indicated by the authors of this last study, there exists an ontogenetic change in the perception of EtOH’s reinforcing properties that coincides with the end of a sensitive period for learning preferences in pups younger than 9 days of age (Sullivan et al., 2000; Roth and Sullivan, 2003). During this sensitive period, certain neurobiological factors (immaturity of the amygdala, low brain corticosterone levels) weaken the capability of the organism to learn certain aversions (Debiec and Sullivan, 2017). Nevertheless, when considering EtOH intoxication there are other important metabolic factors that should be considered when examining the early predisposition to learn conditioned preferences and to exhibit resistance to the drug’s aversive effects. This topic focuses on the motivational role of EtOH’s first metabolite (ACD) during early ontogeny.

## Acetaldehyde Metabolism During Early Ontogeny: Early Motivational Effects of EtOH’s Principal Metabolite

Peripheral and central production of ACD critically modulates sensitivity to EtOH’s motivational properties. In genetically selected animals in terms of alcohol affinity as well as in genetically heterogenous rats and mice, the central production of ACD *via* the catalase system represents a critical factor modulating EtOH’s positive reinforcing effects (Quertemont, 2004; Israel et al., 2015; Peana et al., 2015). The induction of the activity of this enzymatic system potentiates EtOH’s motor stimulating effects (Correa et al., 2001). This stimulatory effect has been considered as an index of positive rewarding central effects of different drugs of abuse (Wise and Bozarth, 1987; Orsini et al., 2004). Correa et al. (2001) showed that in adult rodents pre-treatment with 3-amino-1H,2,4-triazole, a well-known catalase inhibitor, before EtOH administration, significantly reduces EtOH-induced motor stimulation as well as brain catalase activity. In the periphery, EtOH is mainly metabolized into ACD *via* hepatic alcohol dehydrogenase enzymes and the accumulation of the metabolite has been primarily associated with aversive motivational effects (Escarabajal et al., 2003; Sanchis-Segura et al., 2005). Given the hepatic immaturity of fetuses and neonates, the capability to metabolize EtOH into ACD is markedly lower than the one observed during infancy, adolescence and adulthood (Kelly et al., 1987). On the contrary, the activity of the central catalase system negatively correlates with age. The peak activity level of this brain enzymatic system is observed during late gestation and in neonates (Del Maestro and McDonald, 1987). Given this pharmacokinetic profile during early ontogeny, EtOH intoxication implies: (i) a significant accumulation of ACD in the brain; a factor positively correlated with EtOH’s reinforcing effects; and (ii) hepatic immaturity delaying peripheral accumulation of the metabolite which potentially reduces the aversive effects of the drug upon the gastrointestinal system (March et al., 2013b). Considering both processes it appears that, from a metabolic perspective, the balance between central and peripheral ACD production and accumulation during early ontogeny favors positive motivational effects of EtOH.

Different studies based on early EtOH or ACD central administration validate the notion that the metabolite is critical in terms of determining or modulating positive reinforcement. In cesarean delivered rat neonates, only one conditioning trial where an odor was associated with central EtOH administration, was sufficient to generate an olfactory conditioned preference. This effect was completely absent when the catalase system was inhibited with sodium azide (Nizhnikov et al., 2007). Sodium-azide treatment did not affect motor activity when pups were stimulated with the olfactory CS during conditioning or when evaluating nipple attachment at the test; results indicating that neither perception of the CS or the pup’s motor capabilities changed as a function of this drug treatment. Despite these null effects, a follow-up experiment was conducted to test whether the inhibitory effects of sodium azide upon EtOH reinforcement were specific relative to catalase inhibition or if they were merely related to negative consequences upon sensory or learning capabilities of the organism. Rather than employing EtOH as a reinforcer, the study was conducted with the central administration of an endogenous kappa opioid receptor agonist (dynorphin A-13) also known to exert positive reinforcement early in life. The effects of this agonist are independent of metabolic processes corresponding to the catalase system. The study clearly indicated the reinforcing effects of dynorphin that were not affected by inhibition of the catalase system.

Based on these results, studies were performed in cesarean delivered pups intracisternally administered with an EtOH dose (100 mg%) that exerts positive reinforcing effects or an ACD dose (0.35 μ mol) also known to exert psychomotor stimulating effects in adults. Both EtOH and ACD activate the mesolimbic dopamine system which is critical in mediating reinforcing effects of different drugs of abuse (Melis et al., 2007; Diana et al., 2008). Brain administration of the drug or its first metabolite was sufficient to promote the rapid acquisition of conditioned olfactory preferences which was markedly inhibited when sequestering central ACD through the use of d-penicillamine (March et al., 2013a,c). A recent study also showed that maternal administration of d-penicillamine significantly decreases the prenatal reinforcing effects of EtOH when considering different levels of expression of alcohol affinity: (i) attractiveness to EtOH odor as assessed through an odor crawling locomotion neonatal test; (ii) operant responding supported by EtOH reinforcement at postnatal day 5; and (iii) EtOH intake during the second postnatal week (Gaztañaga et al., 2017).

Further support relative to the importance of ACD in the regulation of EtOH’s reinforcing effects was provided by a meta-analytical perspective. The question that guided this approach was the examination of a possible correlation existing between levels of catalase activity and EtOH affinity across ontogeny. As a first step, developmental changes in catalase activity based on average scores (U/mg protein) observed in cerebral hemispheres, striatum, cerebellum and brain stem were taken into account (Del Maestro and McDonald, 1987). As previously mentioned, there is a gradual decrease in the levels of catalase activity as a function of increasing age. Values corresponding to different age groups were linearly correlated with blood EtOH levels derived from alcohol consumption tests performed at similar ages (Truxell and Spear, 2004; Truxell et al., 2007). Spontaneous EtOH intake also decreases gradually across development. This inferential strategy indicated highly significant positive correlations when considering voluntary consumption of 15% v/v (Pearson’s correlation coefficient, *r* = 0.82) or 30% v/v EtOH solutions (*r* = 0.93; March et al., 2013b).

Beyond EtOH’s positive reinforcing effects, is it possible that the antianxiety properties of the drug also participate in the structure of early EtOH-related memories? Antianxiety effects of EtOH have been observed in infant rats. Ultrasonic vocalizations caused by the stress of isolation or derived from a given forced administration procedure (e.g., intraperitoneal, intracisternal or intragastrical) significantly decrease when delivering low to moderate EtOH doses (Pautassi et al., 2007, 2012). Infantile aversive conditioned responses have also been observed to decrease, whenever EtOH is paired with the aversive US; a learning process known as devaluation (Pautassi et al., 2006). Both phenomena argue in favor of early sensitivity to the drug’s antianxiety effects which are similar to those observed when using a GABA-A receptor agonist such as midazolam (Pautassi et al., 2007). Despite these considerations, the involvement of EtOH’s antianxiety properties has not been directly assessed during prenatal or early postnatal life.

Recent studies performed during the stage equivalent to the 3rd human gestational trimester have suggested the implication of EtOH’s antianxiety effects in associative learning processes. The studies have been conducted during postnatal days 3, 5, and 7 in the rat and they were principally aimed at analyzing EtOH’s effects upon neurorespiratory plasticity. During this and earlier stages in development, the immature organism learns an association between different ambient cues and the drug’s depressant effects upon respiration (Cullere et al., 2015; Macchione et al., 2016). We also observed that stressors like maternal deprivation, exposure to a novel context and intragastric or intracisternal administrations had a major disruptive impact upon breathing patterns (Acevedo et al., 2017; Macchione et al., 2018). Recently, utilizing a tactile discrimination procedure we found that intragastric administration of a vehicle solution in maternally deprived pups generated a significant increase in the level of apneic episodes. These disruptions, probably caused by an abnormal level of arousal, were significantly attenuated when EtOH (2.0 g/kg) rather than the vehicle was administered. In this study, two groups of pups were defined by either EtOH or vehicle administration paired with a salient texture across days. A third group received EtOH during the first 2 days of training while the third-day vehicle was administered. Pups in this last group, despite receiving a vehicle, showed similar attenuations of the apneic episodes as those always treated with the drug. This effect appears to indicate a conditioned respiratory response probably associated with the preceding experiences with the antianxiety effects of the drug. It is also important to note that those pups that were always treated with vehicle later exhibited very low levels of preference towards the tactile cue associated with the administration procedure. This apparent conditioned aversion was completely absent in pups previously exposed to the drug (D’Aloisio et al., 2019). Given these results, it can be argued that fetal alcohol programming leading to subsequent EtOH affinity implies the interaction between two non-mutually exclusive motivational properties of the drug: it’s positive as well as its antianxiety effects.

To our knowledge, the role of ACD in the early modulation of EtOH’s anxiolytic effects has not been analyzed. In adult organisms of different altricial species, this phenomenon has been, at most, scarcely analyzed. The results in adults are controversial and dependent upon dosing factors and the site of administration of the metabolite or of drugs that affect the process of EtOH metabolism. Given the marked ontogenetic changes concerning peripheral or central EtOH metabolism, it is also difficult to extrapolate what is known in adults to early ontogenetic stages. Considering this important limitation, the adult literature endorses the anxiogenic effects of the metabolite following its ingestion (Plescia et al., 2015) as well as when the metabolite is intraperitoneally administered (Escrig et al., 2012). When ACD peripheral metabolism was inhibited through cyanamide there were no indications that ACD participates in EtOH-induced anxiolytic effects (Tambour et al., 2005). Focusing on cerebral metabolism of EtOH into ACD, the inhibition of this process or the inactivation of ACD, have a suppressive effect on the anxiolytic actions of EtOH (Correa et al., 2008). As can be observed and considering the limitations of ontogenetic comparisons, there is not enough evidence to understand the role of the metabolite relative to EtOH’s anxiolytic properties.

## Role of The Opioid System in The Modulation of Prenatal EtOH Reinforcement

EtOH induces the release of opioids (Herz, 1997; Gianoulakis, 2001) and the endogenous opioid system plays a major role in EtOH reinforcement (Méndez and Morales-Mulia, 2008). EtOH reinforcing effects are mediated mainly through the stimulation of the mu (MOR) and delta (DOR) opioid receptors (Acquas et al., 1993; Herz, 1997). In infant and adult nondependent rodents, kappa opioid receptors (KOR) appears to mediate the aversive properties of alcohol (Land et al., 2009) and may contribute to EtOH’s anxiolytic effects (Walker and Koob, 2008). The NOP receptor (nociceptin/orphanin FQ peptide) also appears to be critically involved in alcohol affinity. Pharmacological studies indicate that nociceptin and other NOP agonists are effective in reducing EtOH drinking (Martin-Fardon et al., 2010) and reinforcement (Miranda-Morales et al., 2013; Miranda-Morales et al., 2014).

Regarding the effects of prenatal EtOH exposure, general blockade of the opioid system by administration of naloxone to pregnant intoxicated rat dams inhibits the facilitative effect of prenatal EtOH exposure on subsequent EtOH reinforcement in neonates (Miranda-Morales et al., 2010), infants (Chotro and Arias, 2003; Gabriela Chotro and Arias, 2007; Miranda-Morales et al., 2010) and adolescents (Chotro and Arias, 2003). Furthermore, MOR prenatal blockade completely inhibited the facilitative effect on EtOH intake and palatability, while KOR prenatal antagonism only partially reduced the palatability effect (Díaz-Cenzano et al., 2014). Nevertheless, alcohol odor attraction in neonates prenatally exposed to the drug is attenuated by mu or kappa antagonism (Youngentob et al., 2012; Gaztañaga et al., 2015). In summary, this evidence implies activation of the endogenous opioid system following fetal alcohol exposure and the involvement of opioid activity in order for EtOH to function as an effective positive reinforcer during early ontogeny. It is necessary to observe that these effects, as well as the ones that will later be reported, have been encountered when utilizing relatively low to moderate EtOH doses during late gestation. As previously stated, these levels of intoxication fail to exert morphological alterations or to significantly affect the sensory, behavioral or learning capabilities of the developing organism (Molina and Chotro, 1989b; Molina et al., 1989; Lecanuet et al., 1995). In other words, the animal studies under consideration do not meet the criteria of FAS or FASD-like phenotypes as those indicated by Petrelli et al. (2018). Beyond these considerations, it is necessary to observe that animal studies based on chronic and high EtOH doses during gestation have indicated severe alterations in the endorphin system that results in hyperresponsiveness to stressors, a phenomenon that may also lead to heightened EtOH consumption given the antianxiety effects of the drug (Weinberg et al., 1996; Sarkar et al., 2007).

Brief prenatal exposure to moderate EtOH doses significantly reduces opioid peptide concentrations, indicating a possible mechanism by which fetal intoxication can affect future responsiveness towards EtOH (Bordner and Deak, 2015). Other fetal-related studies also indicate selective changes in Methionine-enkephalin (MET-ENK) levels in regions of the mesocorticolimbic and nigrostriatal systems, the hypothalamus and hippocampus showing the involvement of enkephalins in EtOH reinforcement (Abate et al., 2014). MET-ENK levels are specifically changed by fetal intoxication in the above-mentioned central areas during adolescence. These neural enkephalinergic changes modulate later adolescent sensitivity to different motor effects of EtOH (Abate et al., 2017). Interestingly, fetal alcohol experiences also increased levels of mu-opioid receptor transcripts after intake without affecting DOR or KOR receptor transcripts in adolescents (Fabio et al., 2015). MOR mRNA levels revealed differences in infant rats tested in terms of EtOH consumption. This effect was observed when comparing prenatally manipulated subjects (mothers administered with either EtOH or vehicle during late gestation) vs. unmanipulated animals. Consequently, prenatal manipulation promotes changes in MOR mRNA expression (Guttlein et al., 2019). In this study, MOR mRNA levels increased in infant rats following the consumption of the drug. Moreover, prenatal intragastric manipulation during late pregnancy (that can act as a mild stressor) inhibited the increment of MOR mRNA when pups were tested in an EtOH intake test (Guttlein et al., 2019). These studies suggest a key role of MOR and the enkephalinergic systems in EtOH-mediated reinforcement during early ontogeny. In addition, fetal alcohol experiences modulated these opioid sub-systems in terms of further responsiveness to EtOH.

The kappa opioid system is of special interest when regarding fetal alcohol exposure in part because of an established functional switch during ontogeny, from mediating appetitive during early infancy (Petrov et al., 2006) to aversive motivated behaviors (Shippenberg and Herz, 1986; Bals-Kubik et al., 1989). Fetal intoxication also seems to affect KOR function and expression. Infants prenatally exposed to EtOH exhibited either no aversion or appetitive responding to KOR activation, while control subjects reacted aversively following KOR activation. Furthermore, following antenatal experience with the drug, synaptosomal KOR expression was down-regulated in brain areas implicated in the motivational effects of the drug such as the nucleus accumbens, amygdala, and hippocampus (Nizhnikov et al., 2014). As previously mentioned, prenatal EtOH may trigger transient alterations in KORs during early ontogeny. These alterations mimic the levels of KOR mRNA expression in nucleus accumbens, infralimbic cortex and ventral tegmental area in adolescents prenatally exposed to the drug (Fabio et al., 2015). Bordner and Deak (Bordner and Deak, 2015) reported a similar transient result: a heightened expression of prodynorphin (PDYN, the gene coding for the pre-protein that yields dynorphins, the endogenous ligands of KOR) mRNA transcript in ventral tegmental area in rats prenatally exposed to the drug at postnatal day (PD) 4 but not at PDs 8 or 12.While the underlying mechanisms for these effects are not yet clear, it has been recently reported an increase in mRNA levels of KOR and PDYN in the ventral tegmental area of infant and adolescent rats prenatally exposed to EtOH. Epigenetic modifications seem to explain the changes in gene expression since these effects were associated with a reduction of DNA methylation at PDYN and KOR gene promoters (Wille-Bille et al., 2018). Alternative studies also showed that gene expression from the hypothalamus of rat pups (unmanipulated during gestation) exhibited a down-regulated expression of PDYN mRNA and up-regulated mRNA expression of KOR when the first experience with EtOH was defined by infantile consumption of the drug (Guttlein et al., 2019). These results support the hypothesis of Walker and Koob (2008) that repeated EtOH exposure activates an anti-reward system that sensitizes the organism to stress and anxiety-related stimuli and, in turn, promotes a particular sensitivity to EtOH’s anxiolytic effects.

Further research is required to investigate the interconnection between prenatal ACD and the opioid system. D’Addario et al. (2008) reported changes in the expression of the opioid receptors and the precursors of their ligands in response not only to EtOH but also to ACD exposure in human neuroblastoma cells. ACD represents the possible candidate by which EtOH increases the release of β-endorphin which, in turn, can modulate the activity of other neurotransmitter systems such as mesolimbic dopamine (Font et al., 2013). In fact, it has been demonstrated that the opioid system participates in ACD-induced increments in dopaminergic neuronal activity (Fois and Diana, 2016). Specifically, opioid antagonists (naloxone and naltrexone) were found to abolish ACD-induced increase in the firing rate and bursting activity of the dopaminergic neurons of the ventral tegmental area.

The hypothesis that the interaction between ACD and the opioid system in the early regulation of EtOH affinity is also supported, at least indirectly, by preclinical approaches where EtOH maternal intoxication occurred during late prenatal life. As stated, this procedure results in subsequent increases in drug palatability and EtOH intake (Schaal et al., 2013); effects that are eliminated when the state of intoxication is preceded by the administration of the opioid antagonist naltrexone (Miranda-Morales et al., 2014). Interestingly, when utilizing the same animal model but, in this case, sequestering ACD *via* D-penicillamine in fetuses under the state of EtOH intoxication, completely eliminates later affinity for the drug (March et al., 2013c).

## Human Studies Endorsing Fetal Alcohol Programming of Subsequent Alcohol Affinity

When considering human-based literature it is also clear that the fetus processes non-biological volatile substances present in the amniotic fluid. For example, when anise is incorporated in the maternal diet during the last 2 weeks of pregnancy, infants exhibit a stable preference for this odorant while non-exposed controls rather exhibit aversive responding (Schaal et al., 2000). There are also indications that during neonatal life the smell of the amniotic fluid is more effective in guiding nipple attachment even when compared with the natural scent of the mother’s breast (Varendi et al., 2010). When considering the process of lactation, the excellent work of Mennella and Beauchamp (1991, 1993) and Mennella (1998) has demonstrated that human babies not only process minimal amounts of alcohol in maternal milk but also that this experience enhances alcohol odor preferences.

The first human study that was conducted relative to EtOH exposure during fetal life and later neonatal alcohol recognition and discrimination was performed in the 2,000 year (Faas et al., 2000). It is important to note that in this study none of the participants exhibited excessive EtOH intake patterns during pregnancy and all the neonates under evaluation (postpartum age: 24–48 h) were considered as completely healthy as a function of the pertinent clinical and biochemical analyses. Mothers were classified as infrequent or moderate drinkers. This last group was constrained to mothers that drank at least once a week and their consumption scores per occasion averaged approximately 22 g of 190 proof alcohol. Newborns were sequentially stimulated with a cotton swab placed close to the nostrils containing 0.16 g of alcohol or a similar amount of a novel scent (lemon). Head and body movements were video recorded. No differences emerged when contrasting motor activity patterns elicited by lemon odor as a function of maternal alcohol drinking patterns. When employing alcohol odor, babies born to moderate drinkers displayed heightened body, head and facial movements. Subsequently, a new study was conducted using similar experimental procedures and criteria but in this case, the Neonatal Facial Action Coding system was employed (Faas et al., 2015). Facial expressions indicative of appetitive (suckling, smiling and tongue protrusion) or aversive (gaping, brow and nose wrinkling and eye blinking) facial expressions were recorded by blind experimenters. When stimulated with alcohol odor, newborns delivered by moderate drinkers exhibited significantly higher frequencies of appetitive facial responsiveness (particularly when considering tongue protrusion) when compared to babies representative of abstemious or infrequent drinkers. From a correlational perspective the overall amount of alcohol consumed during pregnancy positively and significantly correlated with appetitive reactivity. These results have been recently validated and extended in a clinical investigation where binge drinkers during pregnancy were also included (Anunziata et al., 2019 under revision). Babies born to these mothers were the ones exhibiting the highest levels of appetitive facial expressions, coupled with diminished aversive responding when confronted with alcohol odor. Once again, differential patterns of alcohol use during pregnancy had no effect when newborns were exposed to a novel scent. In summary, moderate or relatively high levels of alcohol intake during pregnancy promote EtOH odor recognition, discrimination and heightened hedonic responses in human newborns.

Does preference for EtOH’s sensory attributes as a function of prenatal exposure to the drug persists in subsequent human ontogenetic stages? The first observation that appears pertinent is that 6-16-year-old children or adolescents heavily exposed to alcohol *in utero* (at least four drinks per occasion at least once a week or 14 drinks per week during gestation) exhibit poor performance in the San Diego Odor Identification Test. None of these subjects was diagnosed with FAS and interestingly, the evaluation did not include EtOH odor (Bower et al., 2013). Despite these olfactory deficits derived from EtOH intrauterine exposure, young adults (18–19 years old) born to mothers that drank at least two standards drinks per day during pregnancy, exhibit heightened relative ratings of pleasantness for alcohol odors (Hannigan et al., 2015). More specifically, higher levels of prenatal alcohol exposure were related to higher ratings of pleasantness for the scent of the drug. The importance of this result is highlighted when considering several factors systematically controlled by the authors of the study. Among others, the study is characterized by detailed information relative to maternal drinking patterns based on beverage type, specific drinking habits, binge drinking episodes, number of standard drinks at particular times of the day and days of the week when the drug was consumed. Participants (young adults with differential levels of prenatal EtOH exposure) were assessed in terms of current risk alcohol use while other control variables were also taken into account (e.g.,: home environment, parenting quality, maternal age at the time of the first prenatal visit, IQ, education, and prenatal exposure to other drugs of abuse such as nicotine, marijuana, and cocaine). When considering the olfactory assessment procedures, participants were evaluated using the “Bottle Test” based on studies performed by Schmidt and Beauchamp (1988) and Mennella and Garcia (2000) which allows the evaluation of olfactory identification in conjunction with the possibility of determining levels of pleasantness elicited by a given odor stimulus. The University of Pennsylvania Smell Identification Test (Doty et al., 1984a,b, Doty et al., 1989; Doty and Agrawal, 1989) was also employed to control possible olfactory dysfunctions as anosmia or mild, moderate, or severe smell loss. The main finding related to the significant association existing between prenatal alcohol exposure and alcohol odor preference endorses the hypothesis of prenatally acquired memories that are retained for long periods of time despite a considerable number of interceding postnatal experiences.

From an epidemiological perspective, approximately a decade ago, Foltran et al. (2011) systemically reviewed the literature linking prenatal maternal drinking and alcohol use and abuse during postnatal life. Seven studies were taken into account; two of them were conducted in Australia (Alati et al., 2006, 2008a,b) while the remaining investigations were performed in the USA (Baer et al., 1998, 2003; Griesler and Kandel, 1998; Yates et al., 1998; Barr et al., 2006). All of these studies with the exception of the one conducted by Yates et al. (1998; case-control investigation) were based on prospective cohort analysis. Evaluations relative to EtOH affinity focused on different dependent variables; among others, children’s drinking patterns at age 14 and onset of alcohol disorders during adolescence and young adulthood. Different covariates were taken into account (e.g., family and maternal history of psychopathological disorders, prenatal consumption of other drugs of abuse, socioeconomic and educational as well as different developmental parameters at birth and during the childhood of the participants under evaluation). As explicitly mentioned in Foltran’s review (Foltran et al., 2011) the overall results of these epidemiological studies “… seem to provide support for a biological origin of some cases of early drinking through a ‘programming’ effect on the brain’s natural reward circuitry. They confirm emerging evidence pointing to *in utero* alcohol exposure, at least at high doses, in the development of addictions.”

Recent epidemiological studies have also strengthened the hypothesis stating that intrauterine EtOH exposure leads to later alcohol use and abuse. In a prospective longitudinal study conducted by Goldschmidt et al. (2019), offspring were evaluated from birth to young adulthood as a function of maternal consumption during the 1st, 2nd and/or 3rd trimester of pregnancy. Relative to higher levels of drinking and Alcohol Use Disorders by age 22, the study concludes that prenatal exposure to even one drink per day significantly augments these problematic consequences. In Sweeden, it has also been observed that individuals diagnosed with FAS are more likely to be hospitalized for alcohol abuse disorders (9% vs. 2% corresponding to healthy control subjects; Rangmar et al., 2015). In a sample of a low-income African-American population in the USA, there have also been indications that prenatal alcohol exposure, especially in young adult males, leads not only to legal difficulties but also to heightened alcohol use. In this study, higher levels of alcohol use were encountered in prenatally exposed subjects that do not exhibit physical or cognitive deficits. When contrasting these results with those reported for example by Rangmar et al. (2015), it appears that alcohol drinking patterns and alcohol use disorders dependent upon gestational exposure to the drug are also modulated by different intervening variables. Ethnicity, alcoholism’s genetic predisposition, socioeconomic and educational status, sensitivity to EtOH’s disruptive effects upon externalizing traits (e.g., low shyness, hyperactivity, attention deficits and conduct problems in childhood and early adolescence) should not be dismissed when determining alcohol use and abuse disorders in fetuses exposed to the drug (Kendler et al., 2013).

In agreement with Foltran et al. (2011), the human literature is congruent with those preclinical studies previously described relative to the concept of fetal alcohol programming of subsequent alcohol affinity. In prior sections, we have emphasized functional sensory and learning capabilities (sensitization to EtOH’s motivational effects and associative learning processes) of the unborn organism that generate specific alcohol-related memories. These processes in no way exclude alternative or complementary mechanisms that may also intervene in the early generation of alcohol programming. One of these mechanisms is related to the dysregulation of the hypothalamic-pituitary-adrenal axis of animals and humans prenatally exposed to the drug (Hellemans et al., 2010) that eventually leads to anxiety, depression, conduct disorders and emotional disorders (Easey et al., 2019). The comorbidity between these disorders and alcohol use and abuse is well known (Kingston et al., 2017; Oliveira et al., 2018).

## Concluding Remarks

The present review focuses on the functional capabilities of the unborn organism that are recruited following alcohol maternal consumption. Both, the animal and human literature consistently demonstrate that fetuses acquire information of EtOH’s chemosensory attributes even when minimal amounts of the drug stimulate olfactory or gustatory receptors. Prenatal exposure to moderate or even high EtOH doses has also been observed to sensitize the organism to the drug’s motivational properties; particularly its positive reinforcing effects or its anxiolytic effects modulated by the endogenous opiate system. The central accumulation of EtOH or of its principal metabolite (ACD) exert unconditioned motivational effects that are rapidly associated with the drug’s sensory cues leading to long-lasting memories that impact upon later alcohol affinity even in terms of generating patterns of drug abuse. As discussed, the process of Fetal Alcohol Programming might occur independently from the teratological effects of the drug.

Fetal Alcohol Programming should broaden our knowledge of potential alterations that are still not taken into account in the diagnostic criteria of FASD. Behavioral signs of Fetal Alcohol Programming have been already detected through relatively simple neonatal techniques developed for altricial mammals including humans (Abate et al., 2008; Faas et al., 2015). These considerations coupled with the ample range of physical, behavioral and cognitive deficits caused by intrauterine EtOH exposure should emphasize the urgent need for more profound and ample primary prevention strategies of the consequences of fetal alcohol exposure. This last statement is even more relevant when considering at least three factors: (i) Internationally, approximately 10% of women consume alcohol while pregnant. One of every 67 of these women delivers a child diagnosed with FAS (Popova et al., 2017); (ii) fertile adolescents and young adults constitute a population segment particularly vulnerable when considering alcohol use and abuse that increases the risk of delivering a child with FASD or programmed in terms of alcohol affinity; and (iii) Re-exposure to the drug during lactation or subsequent stages in development (childhood or adolescence) also constitutes a risk factor in terms of potentiating the imprinting-like phenomenon here referred as Fetal Alcohol Programming [lactation: (Pepino and Mennella, 2004); (Pueta et al., 2008); infants: (Culleré et al., 2014); adolescents: (Eade and Youngentob, 2010)].

## Author Contributions

JM, RM-M, and PA conceptualized and designed the revision and drafted the sequential versions of the manuscript. GD’A and FA participated in the writing process of the revision as well as in the appropriate selection of the studies under examination.

## Conflict of Interest

The authors declare that the research was conducted in the absence of any commercial or financial relationships that could be construed as a potential conflict of interest.
